# Public Stigma against People with Mental Illness in Jimma Town, Southwest Ethiopia

**DOI:** 10.1371/journal.pone.0163103

**Published:** 2016-11-28

**Authors:** Yared Reta, Markos Tesfaye, Eshetu Girma, Sandra Dehning, Kristina Adorjan

**Affiliations:** 1 Program of Nursing, Debire Berhan University, Debire Berhan, Ethiopia; 2 Department of Psychiatry, Jimma University, Jimma, Ethiopia; 3 Department of Preventive Medicine, School of Public Health, Addis Ababa University, Addis Ababa, Ethiopia; 4 CIH^LMU^ Center for International Health, Ludwig-Maximilians-Universität, Munich, Germany; 5 Department of Child and Adolescent Psychiatry, Psychosomatics, and Psychotherapy, Ludwig-Maximilians-University, Munich, Germany; 6 Department of Psychiatry and Psychotherapy, Ludwig-Maximilians-University, Munich, Germany; 7 Institute of Psychiatric Phenomics and Genomics (IPPG), Ludwig-Maximilians-University, Munich, Germany; Maastricht University, NETHERLANDS

## Abstract

**Background:**

Stigma towards people with mental illness (PWMI) can result in low self-esteem and isolation and threaten employment. Therefore, this study aimed to assess the magnitude of public stigma against PWMI and factors associated with it among Jimma town residents.

**Methods:**

A community-based, cross-sectional, descriptive study was conducted in adult residents of Jimma town. Data were collected among 820 randomly selected residents with the interviewer-administered Community Attitudes toward the Mentally Ill (CAMI) scale. Linear regression analyses were performed to identify predictors of stigma against PWMI.

**Result:**

A total of 444 (54%) of the 820 respondents were females, and the mean (SD) age was 35 (8.5) years. The minimum and maximum possible values on each CAMI subscale were 10 and 50, respectively. The respondents had high scores for a stigmatizing attitude towards PWMI across all the subscales, as indicated by the mean (SD) scores: authoritarianism, 27.17 (4.96); social restrictiveness, 32.41 (4.20); benevolence, 35.34 (4.42); and community-based mental health ideology, 33.95 (5.82). Compared to housewives, private organization employees showed more autocratic and socially restrictive views (std. β = 1.12, P<0.01). Single people had a lower social restrictiveness stigma score than married people (std. β = -0.20, P<0.001), and participants’ academic levels correlated inversely with the stigma score (std. β = -0.12, P<0.001). A higher benevolence stigma score was observed among participants with no relationship with PWMI than among those with PWMI in their neighborhood (std. β = 0.08, P< 0.046).

**Conclusion:**

The study revealed that a negative attitude towards PWMI is widespread. Therefore, there is a need to develop strategies to fight the stigma attached to PWMI at the community level.

## Introduction

People with mental illness (PWMI) are likely to experience stigma. Nevertheless, for a very long time the topic of mental health has been hidden behind a cloak of stigma and discrimination [1, 2]. Mental illness and stigma existed long before psychiatry, but on many occasions the psychiatry establishment has not helped to improve stigma towards mental illness [3]. The treatment gap for mental illness is wide throughout the world, which represents an additional burden to those with mental illness [4].

Public stigma is the discrediting response of the general population to people with different conditions [3]. In addition, people with mental illness who experience prejudice may—through their consequent behavior—endorse stereotypical beliefs that people with mental illness are violent, which as a result reinforces the prejudice; prejudice has an evaluative component and also yields emotional responses to stigmatized groups such as anger or fear, which may result in discriminatory act or hostile behavior [5].

Because stigma relates to internal thoughts, it is difficult to take legal action against it. Discrimination, however, refers to actions taken to exclude others because of their perceived differences, and therefore legal protections are possible against discrimination; these protections focus on the behavior itself, rather than on its victims [6, 7].

Both low- and high-income countries have an extensive history of stigma in which PWMI and their families are stigmatized by stereotyping, fear, embarrassment, anger, rejection, and avoidance [1]. The consequences of stigma are extensive and range from effects on quality of life to a level that hinders the effectiveness of many public health programs [8–10]. Stigma and discrimination preclude PWMI from enjoying their social rights and entitlements, and, in so doing, preclude their enjoyment of full citizenship [1, 11]. Consequently, stigma threatens employments and results in low self-esteem, reduced motivation, and discrimination of PWMI by the rest of the community [2].

A review of studies on public attitude to PWMI found that the majority of 61 articles published between 1990 and 2004 investigated stigmatizing attitudes in European and other developed Western nations, and only 15% of the reviewed studies were devoted to non-Western countries [12]. In Africa, studies showed that a high level of public stigma prevails and that there is no strong anti-stigma movement [13–20]. Only a few studies have been performed in Ethiopia; they have shown a prevailing stigmatizing attitude that results in a great burden on patients and their families [21–23].

Many articles argued that there is a need to conduct more mental health stigma studies at the community level [24–27]; unless, while struggling to escape from community imposed stigma, PWMI will face obstacles of visiting psychiatric facilities, which can end up with exacerbation of their state [28–33]. Therefore, this study aimed to determine the magnitude and determinants of stereotypes and prejudices among the public against PWMI and associated factors. Findings from this study could help in planning programs that aim to reduce stigma against PWMI and in bridging the information gap and can be used by scholars in the field as a basis for further robust study.

## Methods

### Ethical statement

Ethical clearance was obtained from the Jimma University Ethical Review Board, and written consent was obtained from the Jimma Town Health Office. Before the actual interview, written informed consent was obtained from all study participants. Data collectors read the consent statement to those study participants who could not read and then obtained their fingerprints as indication of consent. To ensure confidentiality, all interviews were conducted in private places, and data were recorded anonymously.

### Study design and setting

We performed a community-based, cross-sectional study in Jimma town, Southwest Ethiopia, from October to November 2012. Jimma town has 3 districts (woreda) and 13 sub-districts (kebeles). The number of households reported in the town at the time of the study was about 32,191. The 2007 Central Statistical Agency (CSA) census reported the total population of Jimma town to be 120,960 [34], and with a projected growth rate of 4.7 the estimated total population in 2012 was around 152,186. The town has 2 hospitals: a general hospital and a university hospital. Inpatient mental health services are available only at the latter, Jimma University Specialized Hospital.

### Participants and sample size

To determine the sample size, we used the formula for a single population proportion. Because there were no published data on the specific research area, the total sample size was calculated with an assumption of a 50% public stigma level. The minimal difference was taken as 5%, with a 95% confidence interval. Therefore, the estimated sample size was 384. After considering the design effect, the calculated sample size increased to 768. We assumed a 10% non-response rate, so that the final total sample size was 845.

We used multistage probability sampling. From the 13 kebeles (smallest administrative unit) in the town, 5 were selected by a lottery, then the total sample size was proportionally allocated to each kebele. Each sampling unit (household) was selected by systematic random sampling. The first study unit was selected randomly, and if there was more than one study participant (individuals age 18 or above) in a household, a simple random sampling method was used to select one person to participate. If no illegible respondent was found in the selected household, the next household was selected for the study.

### Measurement

The outcome variable was public stigma against PWMI. The exposure variables included sociodemographic and economic factors such as age, sex, religion, ethnicity, level of education, type of occupation, household income, and number of individuals in the household. Other variables such as history of contact with PWMI, history of mental illness, mental health information, and threats from PWMI were included in the study.

Two questionnaires were used. The first comprised questions about sociodemographic variables and other independent variables, and the second was a Community Attitudes toward the Mentally Ill (CAMI) scale to measure the level of public stigma. The CAMI scale includes 40 items rated on a five-point Likert scale from 1 to 5 (1 = strongly agree, 2 = agree, 3 = neither, 4 = disagree, and 5 = strongly disagree) and divided into four subscales: Authoritarianism (AU), Benevolence (BE), Social Restrictiveness (SR), and Community Mental Health Ideology (CMHI) [30]. Authoritarianism refers to a “view of the mentally ill person as someone who is inferior and requires supervision and coercion”; Benevolence is “a humanistic and sympathetic view of mentally ill persons”; Social Restrictiveness covers “the belief that mentally ill patients are a threat to society and should be avoided”; and Community Mental Health Ideology corresponds to “the acceptance of mental health services and the integration of mentally ill patients in the community.” Each subscale consists of ten items and has a minimum score of 10 and maximum score of 50.

Studies in different counties, including African ones, have found the CAMI scale to be reliable [17, 18, 28, 30] and valid, with internal consistency values for the subscales ranging from satisfactory (AU, α = 0.68) to good (BE, α = 0.76, SR, α = 0.80, CMHI, α = 0.88) [30].

The original English version of the CAMI questionnaire was translated into Amharic and Afan Oromo languages and then back translated into English by native speakers and experienced professionals in order to check its consistency. Before the start of the study, consensus on the compatibility of the forward translations to Amharic and Afan Oromo was assured. In this study, the reliabilities of the Amharic version of the CAMI subscales were as follows: AU, α = 0.54; BE, α = 0.46; SR, α = 0.60; CMHI (α = 0.79).

Before the actual data collection, the questionnaire was pre-tested on 5% of a similar population in another kebele, and both the principal investigator and supervisors assessed the clarity, understandability, flow, and completeness of the items and the time needed to complete the questionnaires. This step helped to correct systematic errors, ensured consistency in the flow of items and provided an estimate of the time needed to complete the questionnaire.

### Statistical analysis

The data were checked for completeness and entered into EPI-DATA version 3.1. For analysis, data were exported to SPSS version 20.00 and then cleaned. Reverse coding was performed for negative statements before starting the analysis. Descriptive statistics (frequencies and percentages) were calculated for important variables. Before performing linear regression, the stigma values were checked for normality through histograms and kernel density plots. After performing assumption tests, correlation of continuous independent variables was tested, and an independent sample T-test and one-way ANOVA were used to determine the association of categorical variables with the outcome variable. Finally, variables found to be associated by Pearson correlation, T-test and one-way ANOVA were computed to multiple linear regression to control confounders by using a significance level of <0.05.

## Results

### Sociodemographic characteristics

Of 845 respondents who intended to participate in the study, complete data were obtained for 820 (response rate: 97.2%). Of the 820 respondents, 444 (54.1%) were females. The mean (SD) age of the participants was 34 (14) years. A total of 397 (48.4%) were married, and 330 (40.2%) were single. The major ethnic group was Oromo (n = 218, 26.6%), followed by Amhara (n = 208, 25.4%), and the majority of the participants were Ethiopian Orthodox Christian (n = 432, 52.7%), followed by Muslims (n = 236, 28.8%).

Regarding the educational status of the respondents, 362 (44.1%) had attended secondary school; and 255 (31.1%), college or university. The majority were housewives (n = 184, 22.4%) or students (n = 169, 20.6%). The mean (SD) estimated monthly household income was 1884 (1498) ETB, and the mean (SD) family size in a household was 5 (2) (Table 1).

**Table 1:** Sociodemographic characteristics of respondents in Jimma town, Southwest Ethiopia, November 2012.

Variables		N	%
**Sex**	Female	444	54.1
	Male	376	45.9
**Marital status**	Married	397	48.4
	Single	330	40.2
	Widowed	65	7.9
	Divorced	28	3.4
**Ethnicity**	Oromo	218	26.6
	Amhara	208	25.4
	Kefa	129	15.7
	Gurage	103	12.6
	Dawro	91	11.1
	Others✵	71	8.7
**Religion**	Orthodox	432	52.7
	Muslim	236	28.8
	Protestant	110	13.4
	Catholic	31	3.8
	Others ◆	11	1.3
**Educational Level**	Secondary	362	44.1
	College or university	255	31.1
	Primary	113	13.8
	Unable to read & write	46	5.6
	Able to read & write only	44	5.4
**Occupation**	Housewife	184	22.4
	Student	169	20.6
	Employee in private organization	136	16.6
	Merchant	131	16.0
	Government employee	121	14.8
	Retired	41	5.0
	Others■	38	4.6

Note:

^◆^ Pagan, Jehovah

^✵^ Tigrea, Wolayta, Yem, Siltea, Kulo, Gamo

^■^ Farmer, no job, preacher, house servant

### Exposure to PWMI, and history of mental illness among respondents

Among the total of 820 respondents, 703 (85.7%) had encountered PWMI, and of those a large proportion (47%) reported that PWMI lived in their neighborhood; 27.4% reported that they had no relation to PWMI (i.e. encountered PWMI only on the streets). One-third of the respondents (33%) reported that they had been threatened or attacked by PWMI, and 61% had witnessed others being threatened or attacked. A small proportion (6.2%) of the respondents reported that they themselves had suffered from mental illness at least once in their life time.

### Mental health information

Of the total of 820 respondents, 461 (56.2%) reported that they had heard any information about mental health within the last year, and the remaining 359 (43.8%) reported that they had no information about mental health. Among the respondents who had heard about mental illness in the last year, the leading sources of information were reported to be television (40.1%) and a combination of different sources of information (31%); relatively fewer respondents reported that they had obtained information from health institutions (4.3%) or magazines (4.3%).

### Stigma against PWMI

The internal consistency for the total CAMI scale was found to be α = 0.73; among the sub-scales, CMHI had the highest internal consistency (α = 0.79) and BE the lowest (α = 0.46). The highest stigma score was on the BE subscale (mean 35.34, SD 4.42), and the lowest stigma mean score was on the AU subscale (27.17 ± 4.96)

#### Authoritarianism

The majority of the respondents (74.9%) believed that keeping PWMI behind locked doors is the best way of handling PWMI (A2). More than three-fourths of the participants (86.3%) stated that adults with mental illness needed the same kind of control and discipline as young children (A5), and more than half (56.3%) rejected (“disagree” or “strongly disagree”) the view that virtually anyone can become mentally ill (A10). The majority (88.2%) also agreed with the statement “Mental hospitals are an outdated means of treating the mentally ill” (A9). Even though the mean stigma score was lower on this subscale than on the others, the majority still accepted the negative attitude statements (Table 2).

**Table 2:** Residents’ responses to the items on the Community Attitudes towards the Mentally Ill (CAMI) Authoritarianism subscale, Jimma town, Southwest Ethiopia, November, 2012.

Authoritarianism (AU)	Strongly agree/agree		Neither		Strongly dis agree/disagree	
	N	%	N	%	N	%
One of the main causes of mental illness is a lack of self-discipline and willpower (A1)	235	28.7	18	2.2	567	69.1
The best way to handle the mentally ill is to keep them behind locked doors (A2)	614	74.9	14	1.7	192	23.4
There is something about the mentally ill that makes it easy to tell them from normal people (A3)	407	49.6	14	1.7	399	48.7
As soon as a person shows signs of mental disturbance, he should be hospitalized (A4)	404	49.3	26	3.2	390	47.6
Mental patients need the same kind of control and discipline as a young child (A5)	708	86.3	9	1.1	183	22.3
Mental illness is an illness like any other (A6)	561	68.4	10	1.2	249	30.4
The mentally ill should not be treated as outcasts of society (A7)	402	49.0	12	1.5	406	49.5
Less emphasis should be placed on protecting the public from the mentally ill (A8)	576	70.2	13	1.6	231	28.2
Mental hospitals are an outdated means of treating the mentally ill (A9)	723	88.2	20	2.4	77	9.4
Virtually anyone can become mentally ill (A10)	347	42.3	11	1.3	462	56.3

The multiple linear regression showed that occupation significantly predicted authoritarianism: government employees had 0.13 higher stigma scores in this subscale than housewives (t (791) = 2.49, P = 0.013), and private organization employees had 0.10 higher scores than housewives (t (791) = 2.04, P = 0.042). These results show that both government employees and employees of private organizations have a more autocratic attitude towards PWMI than housewives. The results of the regression indicated that the predictors explained a significant proportion of variance in the AU score (R^2^ = 0.081, F (26, 791) = 42.64, p < .001) (Table 3).

**Table 3:** Multiple linear regression analysis showing significant predictors of Community Attitudes towards the Mentally Ill (CAMI) subscales, in Jimma town, Southwest Ethiopia, November 2012.

Variables		AU		BE		SR		CMHI	
		β	P value	β	P value	β	P value	Β	P value
**Sex**	Male	0.04	0.287						
**Marital status**	Single	0.03	0.477			-0.20	<0.001*		
	Divorced	0.06	0.106			-0.06	0.122		
	Widowed	0.02	0.569			-0.07	0.053		
**Ethnicity**	Amhara	0.08	0.050					-0.02	0.607
	Gurage	0.08	0.063					0.01	0.716
	Kefa	-0.04	0.388					-0.02	0.707
	Dawro	0.01	0.788					-0.03	0.433
	Others	0.07	0.060					0.10	0.053
**Education**	Unable to read & write	-0.01	0.870			-0.37	0.325	-0.08	0.028*
	Able to read & write	-0.06	0.085			-0.06	0.104	-0.10	0.006*
	Primary	-0.04	0.287			-0.12	0.001*	-0.09	0.022*
	College or university	-0.03	0.429			0.11	0.009*	0.02	0.672
**Occupation**	Government employee	0.13	0.013*	0.10	0.022*	0.08	0.110	0.04	0.412
	Employee in private org	0.10	0.042*	0.11	0.016*	1.12	0.010*	-0.00	0.918
	Merchant	0.01	0.864	0.03	0.956	0.06	0.146	0.02	0.586
	Student	0.08	0.179	-0.24	0.608	0.11	0.049*	0.03	0.466
	Retired	0.01	0.732	0.02	0.534	0.03	0.483	0.00	0.992
	Others	0.01	0.961	0.03	0.384	0.03	0.406	0.02	0.666
Estimated monthly household income		-0.07	0.088						
Number of individuals in the household		-0.06	0.086						
Not encountered someone with mental illness						-0.04	0.048*	-0.71	0.250
Relationship to the person with mental illness	Family Member	0.06	0.138	-0.02	0.617				
	Friend	0.01	0.992	0.04	0.304				
	No relationship	0.07	0.059	0.08	0.046*				
Involved in caring		-0.49	0.188	-0.06	0.121	0.07	0.722	0.85	0.054
Threatened or get hurt						0.04	0.219	0.08	0.027
Witnessed others being threatened/attacked						0.01	0.746		

Note: β indicates standardized coefficients Adjusted R2 for each subscale; AU = 0.051, BE = 0.019, SR = 0.066, CMHI = 0.045

* P = < 0.05

AU: Authoritarian, BE: Benevolence, SR: Social restrictiveness; CMHI: Community mental health ideology

#### Benevolence

The majority of respondents (68.4%) rejected the statement that describes the mentally ill as having been the subject of ridicule for too long (BE1). Although a large portion of the respondents (96%) agreed that they have a responsibility to provide the best possible care for PWMI (BE5), 84.0% believed that increased spending on mental health services is a waste of tax money (BE8). A total of 85.1% of respondents rejected the statement “We need to adopt a far more tolerant attitude toward the mentally ill in our society” (BE3), and more than three-fourths agreed with the statement “It is best to avoid anyone who has mental problems” (BE 10). In general, the majority of respondents agreed with the negative attitude statements (Table 4).

**Table 4:** Residents’ responses to the items on the Community Attitudes towards the Mentally Ill (CAMI) Benevolence subscale, Jimma town, Southwest Ethiopia, November 2012.

Benevolence (BE)	Strongly agree/agree		Neither		Strongly dis agree/disagree	
	N	%	N	%	N	%
The mentally ill have for too long been the subject of ridicule (BE1)	233	28.4	26	3.2	561	68.4
More tax money should be spent on the care and treatment of the mentally ill (BE2)	313	38.2	39	4.8	468	57.1
We need to adopt a far more tolerant attitude toward the mentally ill in our society (BE3)	117	14.3	5	0.6	698	85.1
Our mental hospitals seem more like prisons than like places where the mentally ill can be cared for (BE4)	471	57.4	86	10.5	263	32.1
We have a responsibility to provide the best possible care for the mentally ill (BE5)	787	96.0	4	0.5	29	3.5
The mentally ill don't deserve our sympathy (BE6)	499	60.9	8	1.0	313	38.2
The mentally ill are a burden on society (BE7)	451	55.0	19	2.3	350	42.7
Increased spending on mental health services is a waste of tax dollars (BE8)	689	84.0	9	1.1	122	14.9
There are sufficient existing services for the mentally ill (BE9)	535	65.2	80	9.8	205	25.0
It is best to avoid anyone who has mental problems (BE10)	641	78.2	11	1.3	168	20.5

The two most important variables that significantly predicted benevolence were relationship to PWMI and occupation: government employees had 0.10 higher stigma scores for benevolence than housewives (t (808) = 2.30, P = 0.022), and private organization employees had 0.11 higher scores (t (808) = 2.04, P = 0.016). Considering neighbors as a reference, those who had no relationship to PWMI (i.e. only encountered PWMI on the street) had a 0.08 higher benevolence stigma score than those with PWMI in their neighborhood (t (808) = 2.00, P = 0.046). These findings imply that those who only encountered PWMI on the street were more stigmatizing, i.e. they were less benevolent towards PWMI than those who had PWMI in the neighborhood. Also, both government and private employees are less benevolent towards PWMI. These predictors explained 1.9% of the variance in the BE stigma score (R^2^ = 0.019, F (19, 800) = 2.46, P < 0.001) (Table 3).

#### Social Restrictiveness

The statement “The mentally ill should be isolated from the rest of the community” (SR2) was found acceptable by majority of the respondents (84.8%), and 93.0% of them did not want to live next door to someone who has been mentally ill (SR4). A total of 82.7% of the respondents believed that a woman would be foolish to marry a man who had suffered from mental illness, even though he seemed to be fully recovered (SR3). The majority of respondents (73.7%) believed that anyone with a history of mental problems should be excluded from taking public office (SR5). Only 7.3% of respondents believed that PWMI should not be denied their individual rights (SR6), and 78.3% of the respondents did not agree that PWMI should be encouraged to assume the responsibilities of normal life (SR7). A total of 89.6% of respondents rejected the statement “No one has the right to exclude the mentally ill from their neighborhood” (SR8). In general, most of the residents believed that PWMI are a threat to society and should be avoided, indicating that most of them had socially restrictive view towards the PWMI (Table 5).

**Table 5:** Residents’ responses to the items of the Community Attitudes towards the Mentally Ill (CAMI) Social Restrictiveness subscale, in Jimma town, Southwest Ethiopia, November 2012.

Social Restrictiveness (SR)	Strongly agree/agree		Neither		Strongly dis agree/disagree	
	N	%	N	%	N	%
The mentally ill should not be given any responsibility (SR1)	206	25.1	20	2.4	594	72.4
The mentally ill should be isolated from the rest of the community (SR2)	695	84.8	6	0.7	119	14.5
A woman would be foolish to marry a man who has suffered from mental illness, even though he seems fully recovered (SR3)	678	82.7	22	2.7	120	14.6
I would not want to live next door to someone who has been mentally ill (SR4)	763	93.0	2	0.2	55	6.7
Anyone with a history of mental problems should be excluded from taking public office (SR5)	604	73.7	10	1.2	206	25.1
The mentally ill should not be denied their individual rights (SR6)	60	7.3	1	0.1	759	92.6
Mental patients should be encouraged to assume the responsibilities of normal life (SR7)	162	19.8	16	2.0	642	78.3
No one has the right to exclude the mentally ill from their neighborhood (SR8)	84	10.2	1	0.1	735	89.6
The mentally ill are far less of a danger than most people suppose (SR9)	302	36.8	52	6.3	466	56.8
Most women who were once patients in a mental hospital can be trusted as babysitters (SR10)	518	63.2	30	3.7	272	33.2

Marital status, education, occupation, and encountering PWMI independently predicted social restrictiveness and explained a significant proportion of the variance in the SR stigma score (R^2^ = 0.066, F (18, 801) = 4.24, P<0.001). Marital status was associated with social restrictiveness: the mean SR score among single people was 0.20 lower than among married ones (t (801) = -3.85 P<0.001), indicating that married people have more socially restrictive views towards PWMI than single people.

Social restrictiveness increased with higher educational level: people with elementary school education had a 0.12 lower mean SR stigma score than people with a secondary school education (t (801) = -3.26, P = 0.001), and those who were at college or university had a 0.99 higher score than secondary school students (t (801) = 2.61, P = 0.009).

Occupation significantly predicted social restrictiveness: employees of private organizations had a 0.11 higher mean stigma score than housewives (t (801) = 2.57, P = 0.010), as did students (t (801) = 1.97, P = 0.049). Both students and employees of private organizations in general had a more socially restrictive view than housewives. The results of logistic regression showed that encountering PWMI independently had a significant effect on social restrictiveness, i.e. those who had not encountered PWMI had a 0.04 lower SR stigma score than those who had encountered PWMI (t (801) = -1.98, P<0.048) (Table 3).

#### Community Mental Health Ideology

The statement “Residents should accept the location of mental health facilities in their neighborhood to serve the needs of the local community” (CMHI1) was rejected by 90.9% of residents. Many respondents (85.0%) also did not agree with the statement “The therapy for many mental patients is to be part of a normal community” (CMHI2). A total of 74.5% of respondents had a negative view about providing mental health services through community-based facilities (CMHI3), and a large number (91.5%) rather believed that locating mental health facilities in a residential area downgrades the neighborhood (CMHI10). In this subscale, again the majority of participants tended to hold a negative attitude towards PWMI, but less so than in the AU subscale (Table 6).

**Table 6:** Residents’ responses to the subscale items of the Community Attitudes towards the Mentally Ill (CAMI) subscale Community Mental Health Ideology, in Jimma town, Southwst Ethiopia, November 2012.

Community Mental Health Ideology	Strongly agree/agree		Neither		Strongly disagree/disagree	
	N	%	N	%	N	%
Residents should accept the location of mental health facilities in their neighborhood to serve the needs of the local community (CMHI1)	64	7.8	11	1.3	745	90.9
The best therapy for many mental patients is to be part of a normal community (CMHI2)	101	12.3	22	2.7	697	85.0
As far as possible, mental health services should be provided through community-based facilities (CMHI3)	186	22.7	23	2.8	611	74.5
Locating mental health services in residential neighborhoods does not endanger local residents (CMHI4)	300	36.6	17	2.1	503	61.3
Residents have nothing to fear from people coming into their neighborhood to obtain mental health services (CMHI5)	524	63.9	17	2.1	279	34.0
Mental health facilities should be kept out of residential neighborhoods (CMHI6)	261	31.8	6	0.7	553	67.4
Local residents have good reason to resist the location of mental health services in their neighborhood (CMHI7)	345	42.1	54	6.6	421	51.3
Having mental patients living within residential neighborhoods might be good therapy but the risks to residents are too great (CMHI8)	369	45.0	13	1.6	438	53.4
It is frightening to think of people with mental problems living in residential neighborhoods (CMHI9)	547	66.7	23	2	250	30.5
Locating mental health facilities in a residential area downgrades the neighborhood (CMHI10)	750	91.5	8	1.0	62	7.6

In the logistic regression, only education had an independent effect on community mental health ideology; it explained 4.5% of the variance in the stigma score of the CMHI subscale (R^2^ = 0.045, F (19, 800) = 3.033, P< 0.001). Those who were unable to read or write had a CMHI stigma score 0.08 lower than those who had attended secondary school (t (800) = -2.20, P< 0.028), and those who were only able to read and write had 0.10 lower score (t (800) = -2.78 P<0.006). Respondents with only primary school education have 0.09 less score of community mental health ideology than those of secondary school educational level (t (800) = -2.30, P = 0.022). In general, relatively least educated have better community mental health ideology than that of well educated. (Table 3).

## Discussion

The current study attempted to determine the magnitude of public stigma against PWMI and factors associated with such stigma. Stigma was assessed by the Community Attitudes towards the Mentally Ill (CAMI) scale, which has four subscales: Authoritarian (AU), Benevolence (BE), Social Restrictiveness (SR), and Community Mental Health Ideology (CMHI).

Similar to studies performed in Southern Ghana [17], Nigeria [18], India [31], and Taiwan [29], negative views were prevalent in all of the CAMI subscales; in contrast, however, our study found few relative variations among the mean scores of the subscales. A higher mean stigma score was found for BE and CMHI; this finding was similar to the study performed in Southern Ghana [17], but the highest stigma scores recorded in the other studies were for the subscales AU and SR [18, 29, 31]. The finding may differ from that of the Indian study [31] because of its very small sample size (n = 100). The difference from the Nigerian [18] and Taiwanese [29] studies could be a result of the different study population used (study participants in the Nigerian study were teaching hospital senior staff members, and the Taiwanese study used telephone interviews). Our finding may imply that residents of Jimma town are less benevolent and community mental health-oriented and also less authoritarian and socially restrictive than the participants in the other studies [18, 31].

The present study found that residents of Jimma town have an autocratic attitude towards PWMI, shown by the findings that the majority of the respondents believe that keeping PWMI behind locked doors is the best way to handle them (74.9%) and that adults with mental illness need the same kind of control and discipline as young children (86.3%). This finding is similar to that of the Indian study mentioned above [31]. It implies that residents of Jimma town prefer aggressive treatment of PWMI, perhaps because the majority (85.7%) of them had encountered PWMI, 33% of them had been threatened or hurt by PWMI, and 61% had witnessed others being hurt or threatened. Thus, many had experienced aggression in one way or another, which may create fear. Participants may have preferred aggressive treatment of PWMI to protect themselves from impending danger.

More than half of the participants (56.3%) rejected the view that virtually anyone can become mentally ill, which is consistent with findings from Southern Ghana [17], South Africa [20], and India [31]. This may imply that the residents of Jimma town have poor knowledge on perceived causes of mental illness and treatment options; this hypothesis is supported by the finding that 43.8% of the respondents had no information about mental health. Regarding a benevolent view, almost all respondents were of the opinion that they have a responsibility to provide the best possible care for PWMI, a finding that is similar to the study performed in Southern Ghana [17]. Similar to the Indian study [31], more than three-fourths of the respondents considered that it is best to avoid anyone who has mental problems and believe that PWMI do not deserve their sympathy [31]. This implies that respondents place less emphasis on care and choose rather to avoid PWMI than to provide care. This could be a result of poor information on the nature of mental illness and might be also a reaction to the fear of aggression from PWMI.

The majority of respondents had a socially restrictive view towards PWMI. Most respondents said that they do not want to live next door to someone who has been mentally ill and are against a woman marrying a man who has fully recovered from mental illness. This finding is similar to that of two studies performed in Nigeria [16, 18]. Only a minority of the respondents believe that PWMI should not be denied their individual rights, and they also believe that excluding a mentally ill person from the community is not wrong and is permitted. Similar to the studies performed in India [31] and Southern Ghana [17], rather than helping PWMI to assume a normal life and public responsibility, the majority of the respondents would rather exclude and discourage PWMI. Overall, the residents are likely to deny patients their rights, not allow them to take on responsibility, and prohibit marriage and living together with PWMI. These findings may be due to the fact that the majority of the respondents had encountered PWMI on the street, which may have made them think that mentally ill people are unable to look after themselves and are dangerous.

The location of mental health services is challenged by the majority of the respondents in that the statement “Residents should accept the location of mental health facilities in their neighborhood to serve the needs of the local community” was rejected by 90.9% residents. A total of 85% of respondents do not believe that the therapy for patients with mental illness should be part of a normal community. This implies that the residents do not accept that it is helpful for patients to stay in the community, which might also be an additional reason why they prefer to keep the PWMI out of the community. Nearly three-fourths of respondents have a negative view about the provision of mental health services through community-based facilities, which may represent an obstacle when attempting to integrate mental health services at the community level. A large percentage of respondents (91.5%) believe that locating mental health facilities in a residential area downgrades the neighborhood; this finding is consistent with the Nigerian study [18]. This can be explained in a similar way to the previous finding, i.e. respondents prefer to exclude PWMI from the community. The observed agreement with two apparently contradictory statements in different parts of the CAMI scale could have been due to the acquiescent response style of “yay-saying” in the local culture [35].

The study found that highly educated people are more socially restrictive than less educated people. This might mean that better educated people have higher expectations of social responsibility than illiterate people and therefore do not trust PWMI, a finding that is consistent with that of the study performed in Taiwan [29]. Those respondents who attended only primary school and below were less community mental health oriented than those who had attended secondary school, i.e. the latter approved the integration of PWMI and mental health services at the community level more than people with less education; this finding is consistent with the study performed in Southern Ghana [17].

Occupation was associated with scores on all subscales except for CMHI, in which government and private organization employees were less benevolent and more authoritarian than housewives. We found that housewives held more humanistic and sympathetic views towards PWMI; this may be because most housewives are mothers, and their maternal instinct may contribute to their caring and sympathetic tendencies. The participants who were students and private organization employees were more socially restrictive than the housewives, which can be explained in the same manner as the issue of less authoritarianism and benevolence. The studies in Nigeria [18] and India [31] mentioned above did not find that occupation was associated with any of the subscales. This may be because of the different study participants: almost all of the participants in the Nigerian study were medical workers, and the Indian study included only 100 rural residents. In contrast, our study participants were from an urban setting.

Respondents who had PWMI in their neighborhood were more benevolent than those who had no relationship to PWMI, which may mean that those who are more familiar with PWMI are more caring and sympathetic towards them than those who are not familiar with them. This finding is similar to that of a study performed in Toronto, which showed that familiarity with PWMI is associated with a low level of stigma [30].

Having information on mental health was not found to be significant in any of the subscales. However, this finding does not imply that having such information could not reduce the level of public stigma, because our study only asked whether participants had ever heard any information on mental health during the last 12 months and did not ask for any details (e.g. type of information, duration, frequency etc.). Therefore, this point requires further investigation before any conclusions can be reached.

When compared with the Ethiopia Demographic and Health Survey report of 2011, the educational status of our study participants was higher than that of the average Ethiopian community. This is probably because our study participants were recruited from an urban setting (Jimma town), whose community has much better access to education. This could limit the generalizability of our findings to the entire Ethiopian population, who are mainly rural and consequently tend to have a lower education status [36].

## Conclusions

The findings of this study indicate that residents of Jimma town extensively hold a stigmatizing attitude towards PWMI. Overall, the residents are likely to deny PWMI their individual rights, prevent them from taking on various responsibilities and forbid people from marrying and living together with PWMI. In general, few respondents have information on mental health. In situations like this, mental health facilities are expected to play a great role in enhancing mental health awareness, but in contrast our findings show that health service institutions contribute less to delivering mental health information than other sources.

Occupation was the most important predictor strongly associated with authoritarianism, benevolence, and social restrictiveness: all associated subscales showed that housewives have a positive attitude. A higher level of education is related to more social restrictiveness but a better attitude towards accepting the integration of PWMI and mental health services on the community level. Respondents who had encountered someone with mental illness were more socially restrictive, and those who had PWMI in their neighborhood were more benevolent than those who had no relationship with PWMI. The results as a whole indicated that there is an urgent need to develop strategies to enlighten the public regarding the stigma of mental illness and to foster acceptance of PWMI by the community.
